# Layer-by-Layer Assembly of Hierarchical Cotton-Derived C/Cu_2_O/Na_2_MoO_4_ Composite Fibers as Anodes for Lithium-Ion Batteries

**DOI:** 10.3390/nano16181160

**Published:** 2026-09-15

**Authors:** Shun Li, Jidong Zhang, Shuqi Li, Ying Guan, Guijin He

**Affiliations:** 1 School of Chemical and Biological Engineering, Hangzhou Polytechnic University, Hangzhou 311300, China; 2 Department of Chemistry, Zhejiang University, Hangzhou 310027, China

**Keywords:** layer-by-layer assembly, cotton-derived carbon, sodium molybdate, interfacial engineering, lithium-ion batteries

## Abstract

Sodium molybdate (Na_2_MoO_4_) is attractive for lithium storage because of its multielectron conversion chemistry, but its low electrical conductivity and structural reconstruction during cycling limit active-material utilization and long-term reversibility. Here, hierarchical C/Cu_2_O/Na_2_MoO_4_ composite fibers were constructed by combining a cotton-derived micro/nanofibrous carbon framework with Cu_2_O interfacial modification and electrostatic layer-by-layer (LbL) assembly. The resulting architecture retains the interconnected fibrous structure of the original cotton fibers. A Na_2_MoO_4_-containing active layer is constructed on the Cu_2_O-modified carbon fiber surface, and its loading can be regulated by changing the number of LbL assembly cycles. When evaluated as an anode for lithium-ion batteries, the C/Cu_2_O/Na_2_MoO_4_-50 composite exhibits the best overall electrochemical performance, with an initial Coulombic efficiency of 65.2% and a reversible capacity of approximately 579.3 mAh g^−1^ after 1000 cycles, whereas pristine Na_2_MoO_4_ retains only about 66.7 mAh g^−1^. The hierarchical fibrous carbon network supplies long-range electron conduction, while the micro- and nanoscale fiber surface places the conversion-active phase close to the electrolyte and shortens the Li^+^ transport distance. The Cu_2_O-modified surface provides the interface for subsequent LbL deposition, allowing Na_2_MoO_4_ to remain distributed along the conductive fiber rather than forming an isolated bulk active phase. This work shows that combining biomass-derived structural inheritance with controllable LbL assembly provides a viable route for regulating the integration and utilization of conversion-active phases in lithium storage electrodes.

## 1. Introduction

The increasing demand for higher energy density and faster charging capability has exposed the limitations of conventional graphite anodes, whose theoretical capacity is restricted to 372 mAh g^−1^ [1,2]. Therefore, developing advanced anode systems with high reversible capacity, rapid reaction kinetics, and long-term structural stability has become an important research direction. However, achieving practical improvements requires not only increasing the capacity contribution of active materials but also addressing the challenges associated with sluggish reaction kinetics, interfacial instability, and structural degradation during long-term cycling. Consequently, advanced anode architectures must provide sufficient active material, efficient ion and electron transport, and persistent electrical contact during prolonged cycling [3].

Transition-metal compounds have attracted extensive attention as promising alternatives to graphite because their conversion reactions involve multiple electrons and provide higher theoretical capacities [4,5,6,7]. Among them, molybdenum-based compounds are particularly attractive due to the variable oxidation states of Mo and their multielectron redox processes during lithiation and delithiation. Sodium molybdate (Na_2_MoO_4_), containing Mo^6+^ species, has been explored as a promising conversion-type anodic material because its multistep redox reactions can provide considerable lithium-storage capacity [8]. However, the conversion process involves extensive structural reconstruction, while the intrinsically low electrical conductivity of Na_2_MoO_4_ restricts charge transfer and active-material utilization [9,10,11].

Integrating Na_2_MoO_4_ with conductive carbon materials is an effective approach to improve its electrochemical performance [11,12]. However, the key challenge is not only improving conductivity but also precisely controlling the spatial distribution and loading of conversion-active phases within conductive frameworks. Carbon coatings, carbon nanotubes, graphene frameworks, and other conductive matrices can enhance electron transport and provide buffering space for structural variation during cycling [13]. However, conventional carbon modification methods generally rely on simple mixing or coating processes, which provide limited control over the distribution and loading of active phases. For conversion-type electrodes, increasing active-material loading does not necessarily guarantee improved performance because excessive loading may increase diffusion resistance and hinder charge-transfer processes. Therefore, constructing a carbon-supported electrode with a hierarchical conductive framework and controllable active-phase deposition is highly desirable [14].

Biomass-derived carbon materials are attractive for constructing hierarchical electrode architectures because their intrinsic multiscale structures can be inherited after carbonization [15,16]. Natural cellulose fibers contain interconnected microfibers and nanoscale fibrils, which can be converted into conductive carbon frameworks while preserving the structural characteristics of the original precursors. Such hierarchical carbon structures can provide continuous electron-transport pathways, facilitate electrolyte penetration, and accommodate the mechanical stress generated during electrochemical reactions [17,18]. Cotton, as a typical cellulose-based material, possesses a continuous fibrous network suitable for constructing hierarchical carbon electrodes. Through chemical pretreatment, the surface of cotton fibers can be partially fibrillated while maintaining the microscale framework, generating abundant nanoscale interfaces for active-material deposition. After carbonization, the resulting hierarchical carbon fibers can serve as conductive backbones and structural supports for conversion-type active materials.

Although hierarchical carbon frameworks provide an effective platform for conversion-type active materials, the distribution and loading of Na_2_MoO_4_ on complex fibrous substrates must also be controlled because excessive local deposition can increase transport resistance, whereas insufficient loading limits the contribution of the active phase. Recent electrode-fabrication strategies have enabled active-material control at different structural scales. Atomic layer deposition (ALD) provides nanoscale surface modification with controllable coating thickness, while electrospinning enables the construction of continuous one-dimensional composite networks with regulated component distribution. At the electrode scale, 3D printing allows programmable control of electrode geometry, filament spacing, and active-material loading, and hierarchical 3D electrode design has also been used to distribute active materials throughout high-loading conductive frameworks [19,20,21]. For the irregular cotton-derived fibrous substrate used here, LbL assembly provides a solution-based route to regulate the amount and surface distribution of Na_2_MoO_4_ through sequential adsorption of oppositely charged species [22,23]. In this work, Cu_2_O was introduced as an interfacial functional component, followed by alternating assembly of PDDA and MoO_4_^2−^ species on the hierarchical cotton-derived framework. By varying the assembly cycles, the effect of Na_2_MoO_4_ loading on interfacial kinetics and lithium-storage behavior was examined. This design therefore separates the influence of active-phase loading from major changes in the carbon framework and provides a basis for understanding the relationship between loading, transport, and cycling stability.

## 2. Experimental Section

### 2.1. Preparation of Micro/Nanostructured Cotton Fibers

Cotton fibers were chemically treated through sequential acid and alkali processes to construct a hierarchical micro/nanostructure while maintaining the original fibrous framework. Typically, 10.0 g of cotton fibers were immersed in 50 mL of H_2_SO_4_ solution (2.0 mol L^−1^) and stirred for 2 h, followed by thermal treatment at 80 °C for 8 h to induce partial hydrolysis of cellulose fibers. The treated fibers were repeatedly washed with deionized water until neutral conditions were reached, followed by ethanol rinsing and vacuum drying.

Subsequently, the dried acid-treated cotton fibers were transferred into NaOH solution (4.0 mol L^−1^) and maintained at room temperature for 20 h. After alkali treatment, the fibers were washed with deionized water and ethanol, followed by vacuum drying to obtain micro/nanostructured cotton fibers (MCF). The acid–alkali treatment generated nanoscale fibrillar structures on the surface of the cotton fibers while preserving the microscale fibrous framework.

### 2.2. Preparation of MCF/Cu_2_O Composite Fibers

The MCF/Cu_2_O composite fibers were prepared through an in situ glucose-assisted reduction process. Fresh copper hydroxide species were generated under alkaline conditions and subsequently reduced in the presence of glucose, leading to Cu_2_O deposition on the MCF surface.

Typically, 5.0 g of MCF was dispersed in 50 mL glucose solution (0.1 mol L^−1^) and stirred at room temperature for 4 h. Meanwhile, 5 mL Cu(NO_3_)_2_ solution (2 wt%) was slowly added to 20 mL NaOH solution (10 wt%) under magnetic stirring for 3 min to generate fresh copper hydroxide species. The obtained suspension was then transferred into the glucose-containing MCF dispersion and heated at 80 °C for 6 h.

After the reaction, the product was repeatedly washed with deionized water to remove residual glucose, followed by ethanol washing and vacuum drying overnight. The obtained material was denoted as MCF/Cu_2_O.

### 2.3. Preparation of C/Cu_2_O/Na_2_MoO_4_ Composite Fibers Through Layer-by-Layer Assembly

The C/Cu_2_O/Na_2_MoO_4_ composite fibers were fabricated using an electrostatic layer-by-layer (LbL) assembly strategy. Positively charged PDDA and negatively charged MoO_4_^2−^ species were sequentially deposited onto the surface of MCF/Cu_2_O fibers through alternate adsorption cycles.

Briefly, MCF/Cu_2_O fibers were placed in a filtration device, and 20 mL PDDA solution was added and allowed to adsorb for 10 min under gentle stirring. After removing the excess PDDA solution, the fibers were rinsed with deionized water to eliminate loosely attached polymer chains. Subsequently, 20 mL Na_2_MoO_4_ solution (0.2 mol L^−1^), prepared from Na_2_MoO_4_·2H_2_O, was introduced using the same procedure to deposit negatively charged MoO_4_^2−^ species onto the fiber surface.

The PDDA/MoO_4_^2−^ deposition cycle was repeated for different numbers of assembly cycles to regulate the loading amount of Na_2_MoO_4_. After drying, the obtained composite fibers were carbonized at 500 °C for 6 h under an Ar atmosphere. During carbonization, the cotton-derived framework was converted into conductive carbon while preserving the hierarchical fibrous morphology. Meanwhile, the organic components introduced during assembly were carbonized or removed, leaving the inorganic Na_2_MoO_4_-containing active layer integrated with the carbon framework.

The samples prepared with 30 and 50 LbL deposition cycles were denoted as C/Cu_2_O/Na_2_MoO_4_-30 and C/Cu_2_O/Na_2_MoO_4_-50, respectively.

### 2.4. Material Characterization

The crystalline phases of the prepared samples were identified by X-ray diffraction (XRD, X’Pert PRO, Philips Analytical, Almelo, The Netherlands) using Cu Kα radiation (λ = 0.15418 nm). The diffraction patterns were recorded in the 2θ range of 5–80° with a scanning rate of 10° min^−1^. The structural characteristics of carbon materials were analyzed by Raman spectroscopy (LabRAM HR UV, HORIBA Jobin Yvon, Longjumeau, France) using a 514 nm laser excitation source. The surface elemental composition and chemical states of the samples were examined by X-ray photoelectron spectroscopy (XPS, Escalab Mark II, VG Scientific, East Grinstead, UK) equipped with a monochromated Mg Kα radiation source (hν = 1253.6 eV). All binding energies were calibrated using the C 1s peak at 284.8 eV. The morphology and microstructure of the samples were characterized by scanning electron microscopy (SEM, Hitachi SU-70, Hitachi High-Technologies Corporation, Tokyo, Japan) operated at an accelerating voltage of 20 kV and transmission electron microscopy (TEM, Hitachi HT-7700, Hitachi High-Technologies Corporation, Tokyo, Japan) operated at 100 kV. For SEM and TEM measurements, the samples were ultrasonically dispersed in ethanol to obtain homogeneous suspensions. The obtained dispersions were dropped onto aluminum foil substrates for SEM observation and copper grids for TEM analysis, followed by drying at room temperature.

### 2.5. Electrochemical Measurements

The working electrodes were prepared by mixing the active materials, acetylene black, and poly(vinylidene fluoride) (PVDF) binder with a mass ratio of 8:1:1. The mixture was thoroughly ground and transferred into a weighing bottle, followed by vacuum drying overnight. A small amount of acetone was then added, and the mixture was ultrasonically dispersed for 1 h. Subsequently, N-methyl-2-pyrrolidone (NMP) was introduced to form a homogeneous slurry after continuous stirring for 12 h.

The obtained slurry was uniformly coated onto nickel foam current collectors and dried under vacuum. The electrodes were then pressed at 10 MPa to obtain the final working electrodes. The mass loading of active materials was approximately 1.5 mg cm^−2^. Coin-type half cells were assembled in an argon-filled glove box with both oxygen and moisture levels below 0.1 ppm. Lithium metal foil was used as both the counter and reference electrode, while Celgard 2300 polypropylene membrane was used as the separator. The electrolyte consisted of 1.0 M LiPF_6_ dissolved in a mixture of ethylene carbonate (EC) and diethyl carbonate (DEC) with a volume ratio of 1:1.

The galvanostatic charge–discharge measurements, cycling performance, and rate capability tests were performed on a Neware battery testing system (Neware Technology Co., Ltd., Shenzhen, China). The long-term cycling test was conducted at a constant current density of 0.5 A g^−1^ within the voltage range of 0.01–3.0 V (vs. Li^+^/Li). Cyclic voltammetry (CV) measurements were carried out using a CHI760D electrochemical workstation (CH Instruments, Inc., Austin, TX, USA) at a scan rate of 0.1 mV s^−1^ over the potential window of 0.01–3.0 V (vs. Li/Li^+^). The specific capacities were calculated based on the mass of active materials. Electrochemical impedance spectroscopy (EIS) was conducted on a CHI760D workstation over a frequency range of 100 kHz to 0.01 Hz using an AC perturbation amplitude of 5.0 mV.

## 3. Results and Discussion

**Scheme 1:**
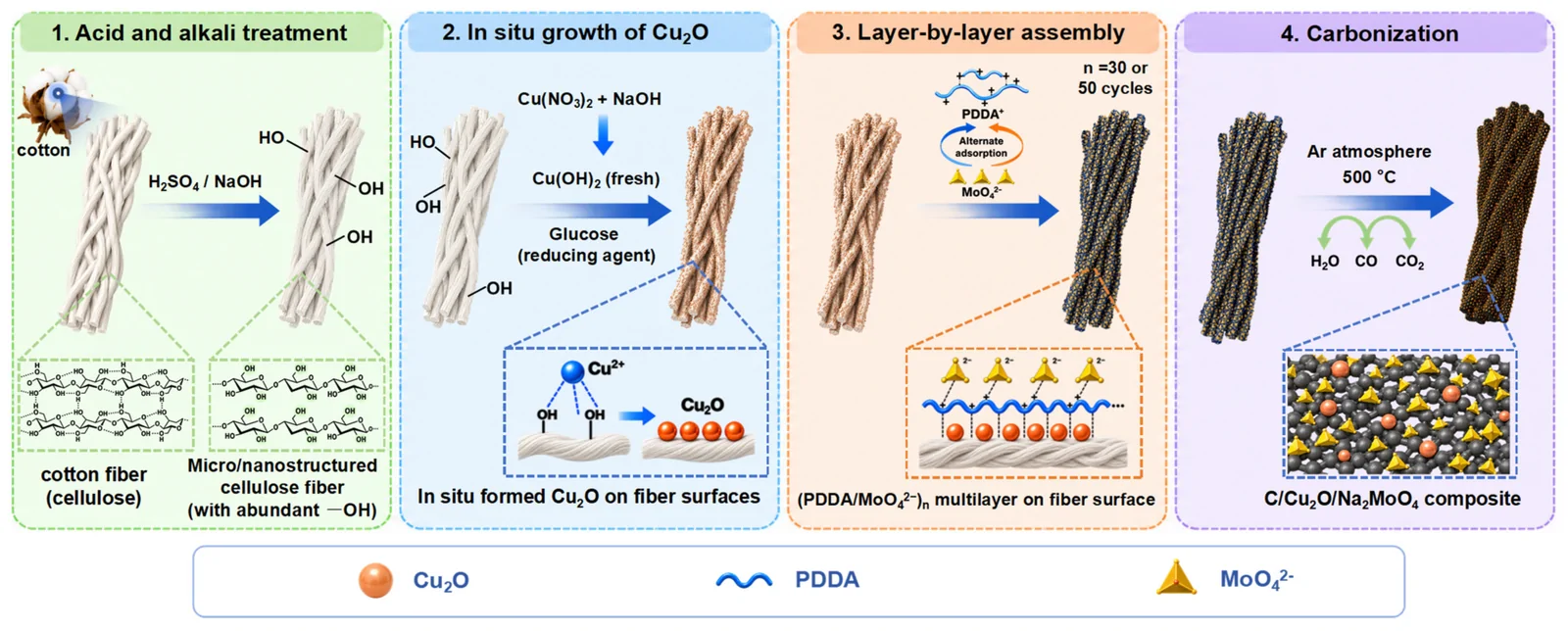
Schematic illustration of the multistep preparation and key chemical transformations involved in the formation of hierarchical C/Cu_2_O/Na_2_MoO_4_ composite fibers.

A hierarchical fiber architecture was constructed by integrating a cotton-derived carbon framework with interfacial Cu_2_O modification and controllable Na_2_MoO_4_ assembly (Scheme 1). Natural cotton fibers were first transformed into micro/nanostructured fibers through acid–alkali treatment, generating abundant nanoscale fibrils while preserving the continuous fibrous framework. At the molecular level, acid hydrolysis partially cleaves the cellulose chains and preferentially removes less ordered regions, while the subsequent alkali treatment disrupts and reorganizes the hydrogen-bonding network, thereby promoting fibrillation and exposing more surface hydroxyl groups. Cu_2_O was subsequently introduced onto the fiber surface. Fresh Cu(OH)_2_ species formed under alkaline conditions were reduced by glucose, resulting in the in situ deposition of Cu_2_O on the hydroxyl-rich cellulose surface. The Na_2_MoO_4_-containing layer was then deposited through alternating adsorption of positively charged PDDA and negatively charged.

MoO_4_^2−^ species driven by electrostatic interactions [24]. By varying the number of assembly cycles (30 and 50 cycles), the amount of deposited Na_2_MoO_4_ was controllably adjusted. During subsequent carbonization, the cellulose framework was converted into carbon while the hierarchical fibrous architecture and surface-deposited inorganic components were retained.

**Figure 1:**
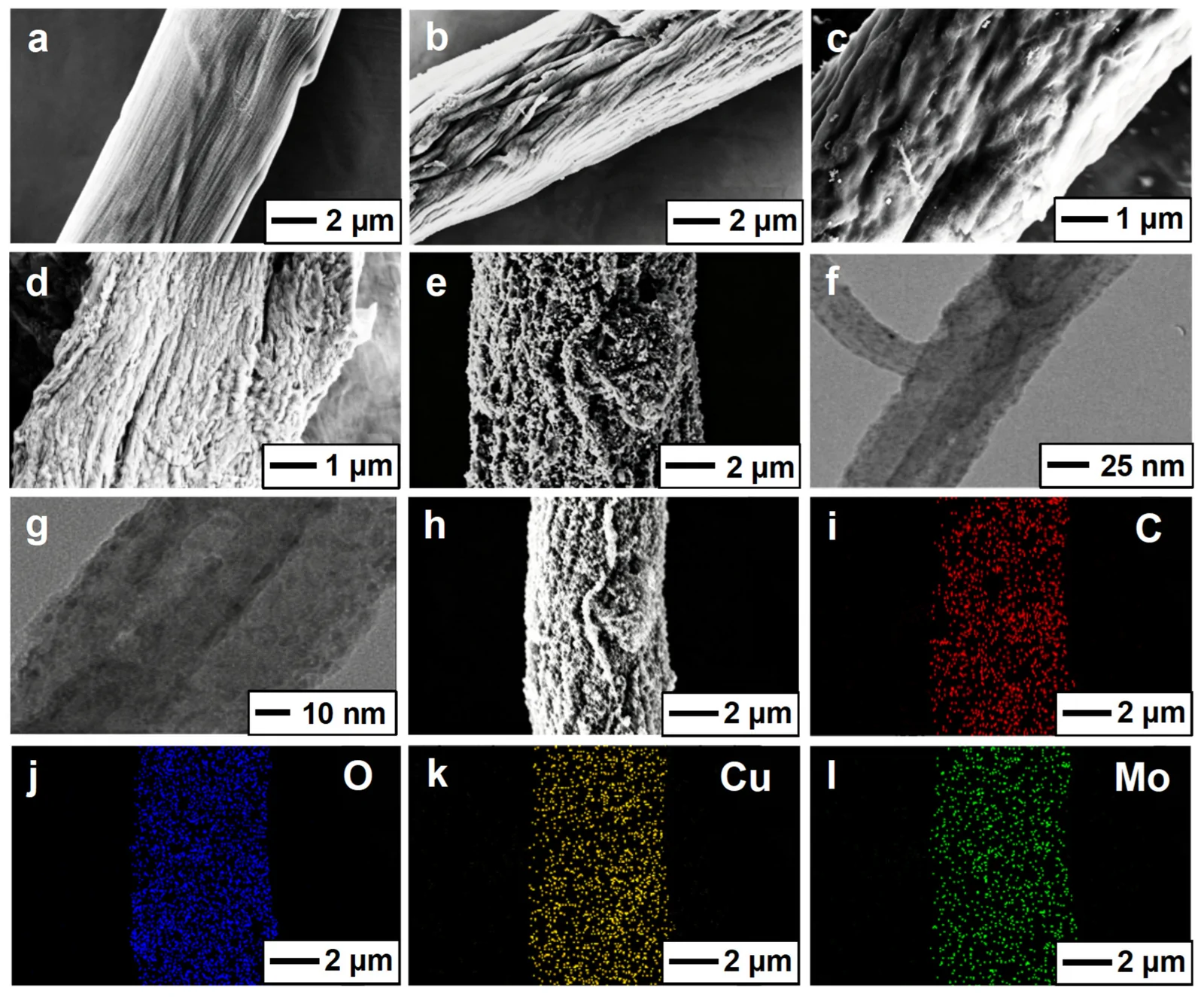
SEM images of (**a**) pristine cotton fibers, (**b**) micro/nanostructured cotton fibers, (**c**) Cu_2_O-modified cotton fibers, (**d**) C/Cu_2_O/Na_2_MoO_4_-30, and (**e**) C/Cu_2_O/Na_2_MoO_4_-50. TEM images of (**f**) C/Cu_2_O/Na_2_MoO_4_-30 and (**g**) C/Cu_2_O/Na_2_MoO_4_-50. (**h**) SEM image and corresponding EDS elemental mappings of (**i**) C, (**j**) O, (**k**) Cu, and (**l**) Mo for C/Cu_2_O/Na_2_MoO_4_-50.

The morphological properties of the composite fibers were investigated by SEM and TEM. As shown in Figure 1a, the pristine cotton fibers exhibit a smooth surface with a continuous micron-scale fibrous structure, and the fiber diameter is approximately 6–8 μm. After sequential acid–alkali treatment, the surface morphology changes significantly (Figure 1b). Numerous interconnected nanoscale fibrils are generated on the fiber surface, while the original macroscopic fiber framework remains intact. This hierarchical micro/nanostructure increases the accessible surface area and provides abundant surface sites for subsequent interfacial modification and active-phase assembly.

After in situ growth of Cu_2_O, the surface morphology of the cotton fibers becomes relatively compact (Figure 1c). Compared with the untreated micro/nanostructured fibers, the fibrillar features remain visible, indicating that Cu_2_O deposition does not destroy the hierarchical framework. Meanwhile, a small number of Cu_2_O nanoparticles can be observed on the fiber surface, which originate from the reduction of copper hydroxide species by glucose during the reaction. The introduction of Cu_2_O provides an inorganic interface for subsequent electrostatic layer-by-layer assembly while preserving the structural advantages of the cotton-derived scaffold. After carbonization, the obtained C/Cu_2_O/Na_2_MoO_4_-30 and C/Cu_2_O/Na_2_MoO_4_-50 composite fibers (Figure 1d,e) maintain the original one-dimensional fibrous morphology and micro/nanostructured surface features. No obvious collapse or severe aggregation is observed after the high-temperature treatment, demonstrating that the hierarchical architecture derived from cotton fibers can be effectively retained during carbonization. This structural inheritance is beneficial for constructing continuous conductive pathways and providing accessible interfaces for electrochemical reactions.

The detailed microstructure of the composite fibers was further examined by TEM. As shown in Figure 1f,g, a surface-deposited inorganic layer can be observed on the surface of the carbon fibers, corresponding to the deposited inorganic component. The thickness of the deposited layer is approximately 30 nm for C/Cu_2_O/Na_2_MoO_4_-30 and around 50 nm for C/Cu_2_O/Na_2_MoO_4_-50. Increasing the number of LbL cycles from 30 to 50 therefore increases the thickness of the deposited surface layer by approximately 20 nm.

To further verify the distribution of different components, EDS elemental mapping was performed on the C/Cu_2_O/Na_2_MoO_4_-50 composite fiber. As shown in Figure 1h–l, the elements C, O, Cu, and Mo are uniformly distributed throughout the selected fiber region, and their spatial distributions closely match the morphology of the carbon fiber. The Mo mapping extends continuously along the fiber surface without obvious localized accumulation, showing that the Mo-containing component is distributed over the carbon fiber rather than concentrated in isolated regions. Semiquantitative EDS analysis was used to estimate the contents of the inorganic components. Assuming that the detected Mo originates predominantly from Na_2_MoO_4_, the Na_2_MoO_4_ content was calculated from the measured Mo content according to the stoichiometric mass ratio between Na_2_MoO_4_ and Mo. On this basis, the estimated Na_2_MoO_4_ contents are 15.3 wt% for C/Cu_2_O/Na_2_MoO_4_-30 and 27.4 wt% for C/Cu_2_O/Na_2_MoO_4_-50. Similarly, the Cu_2_O content was estimated from the measured Cu content assuming Cu_2_O as the dominant Cu-containing phase. Thus, increasing the LbL assembly cycles from 30 to 50 increases the Na_2_MoO_4_ content by approximately 12.1 wt%. Combined with the corresponding increase in surface layer thickness from approximately 30 to 50 nm, these results indicate that the LbL cycle number controls both the amount and thickness of the deposited active phase.

**Figure 2:**
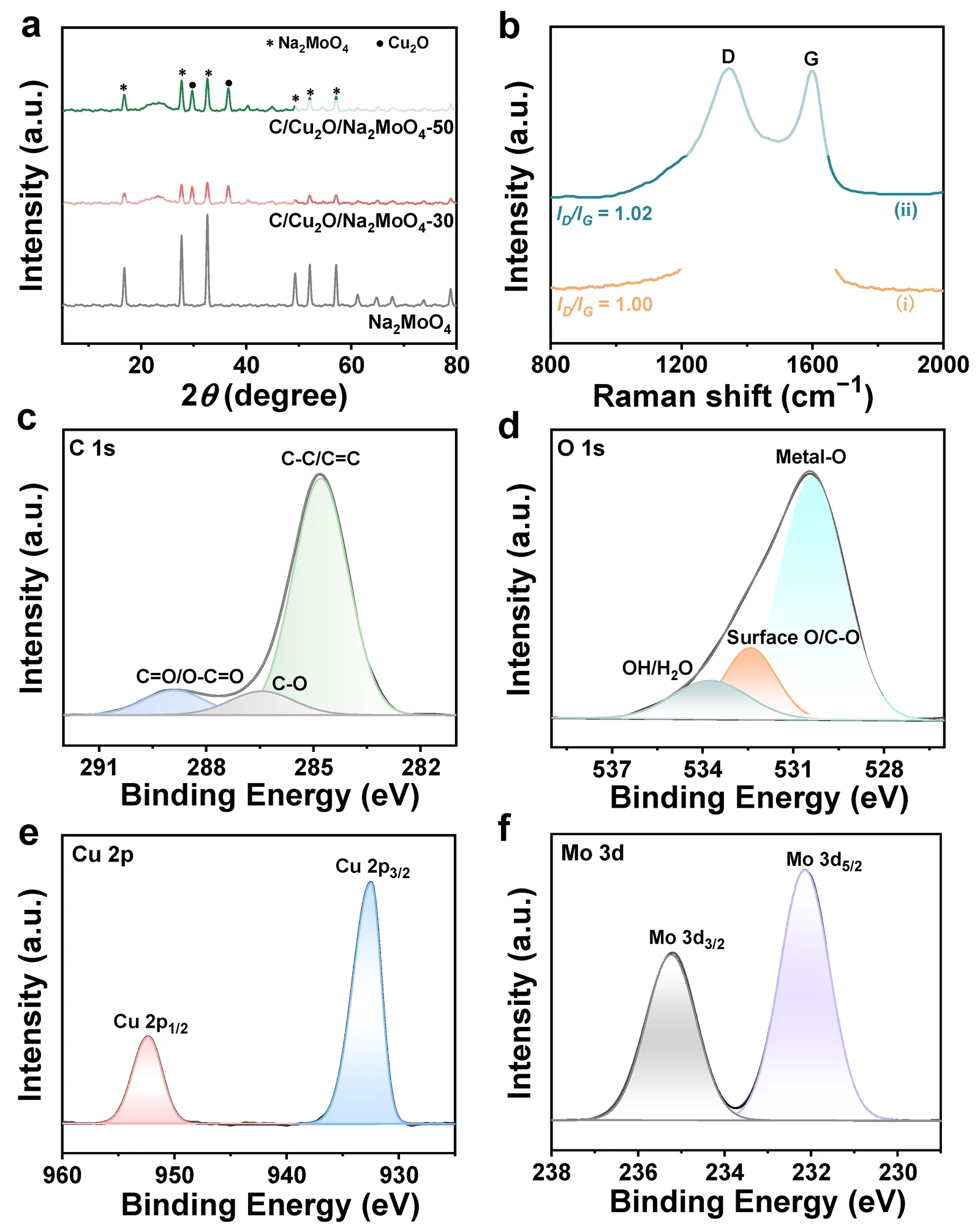
(**a**) XRD patterns and (**b**) Raman spectra of C/Cu_2_O/Na_2_MoO_4_-30 (curve i) and C/Cu_2_O/Na_2_MoO_4_-50 (curve ii). High-resolution XPS spectra of (**c**) C 1s, (**d**) O 1s, (**e**) Cu 2p, and (**f**) Mo 3d for C/Cu_2_O/Na_2_MoO_4_-50 composite.

The crystal structures of the obtained samples were further examined by X-ray diffraction (XRD), as shown in Figure 2a. The diffraction peaks at approximately 16.8°, 27.7°, 32.6°, 49.3°, 52.1°, and 57.1° are assigned to the (111), (220), (311), (422), (511), and (440) planes of cubic *α*-Na_2_MoO_4_ with a spinel-type structure (space group Fd-3m, JCPDS No. 12-0773), respectively. The peaks located at 29.7° and 36.6° correspond to the (110) and (111) planes of cubic Cu_2_O with the cuprite structure (space group Pn-3m, JCPDS No. 05-0667), respectively [9,11,25]. The coexistence of these characteristic reflections confirms the presence of crystalline Na_2_MoO_4_ and Cu_2_O phases in the composite fibers. Compared with C/Cu_2_O/Na_2_MoO_4_-30, C/Cu_2_O/Na_2_MoO_4_-50 exhibits stronger Na_2_MoO_4_ reflections, showing a larger contribution from the crystalline Na_2_MoO_4_ phase after 50 LbL assembly cycles. Meanwhile, a broad diffraction feature centered around 23° is observed in both composite fibers, corresponding to the (002) reflection of carbon. The absence of sharp carbon diffraction peaks suggests that the cellulose-derived carbon is mainly composed of disordered carbon rather than highly graphitized carbon [26].

The carbon structures of C/Cu_2_O/Na_2_MoO_4_-30 and C/Cu_2_O/Na_2_MoO_4_-50 were further investigated by Raman spectroscopy (Figure 2b). Both samples exhibit two typical carbon bands located at approximately 1345 and 1599 cm^−1^, corresponding to the D band and G band, respectively. The D band is associated with structural defects and disordered carbon domains, whereas the G band originates from the in-plane vibration of sp^2^-hybridized carbon atoms [27,28]. The calculated intensity ratios (*I*_D_/*I*_G_) of C/Cu_2_O/Na_2_MoO_4_-30 and C/Cu_2_O/Na_2_MoO_4_-50 are 1.00 and 1.02, respectively. The difference of only 0.02 shows that increasing the LbL assembly cycles does not appreciably alter the degree of disorder of the cotton-derived carbon framework. The principal structural change introduced by increasing the assembly cycles is therefore the amount and thickness of the Na_2_MoO_4_ containing surface layer.

The surface chemical states of the C/Cu_2_O/Na_2_MoO_4_-50 composite fibers were further investigated by high-resolution X-ray photoelectron spectroscopy (XPS). The survey spectrum shown in Figure S1 confirms the presence of C, O, Cu, Mo, and Na elements, indicating the successful integration of carbon, Cu-containing species, and Na_2_MoO_4_ components within the composite fibers. The detailed XPS fitting results, including the binding energies, FWHM values, and fitting constraints for the individual components, are summarized in Table S1.

The high-resolution Na 1s spectrum (Figure S2) contains two components centered at approximately 1071.0 and 1071.55 eV. The component at 1071.55 eV is consistent with Na^+^ species, while the lower-binding-energy component indicates a different local Na environment [29]. The surface Na/Mo atomic ratio determined from the sensitivity-factor-corrected Na 1s and Mo 3d signals is approximately 2.1, close to the stoichiometric value of 2 for Na_2_MoO_4_.

The high-resolution C 1s spectrum (Figure 2c) can be deconvoluted into three characteristic peaks located at approximately 284.8, 286.5, and 288.8 eV. The first two components are assigned to C–C/C=C and C–O species, respectively, whereas the higher-binding-energy component at 288.8 eV is attributed to C=O and O–C=O environments, including possible carboxyl-and ester-type surface groups [26]. The dominant C–C/C=C is consistent with the carbonaceous framework formed after carbonization, while the higher-binding-energy components reflect residual oxygen-containing surface functionalities. The O 1s spectrum (Figure 2d) provides information about the different oxygen chemical environments within the C/Cu_2_O/Na_2_MoO_4_ composite fibers. The peak located at approximately 530.5 eV is associated with lattice oxygen in metal–oxygen bonds originating from the inorganic Cu- and Mo-containing phases. Because the O 1s binding energies of Cu–O and Mo–O species overlap, their individual contributions cannot be reliably separated from the O 1s spectrum alone. The signal centered at around 532.4 eV can be assigned to surface oxygen-containing species, particularly C–O containing groups within the carbon framework. The weak contribution at higher binding energy near 533.8 eV is attributed to physically adsorbed water molecules or hydroxyl groups on the fiber surface. These O 1s features are consistent with the coexistence of lattice oxygen and surface oxygen-containing species in the composite. The Cu 2p spectrum (Figure 2e) displays two main peaks at 932.5 and 952.3 eV, corresponding to Cu 2p_3/2_ and Cu 2p_1/2_, respectively. Only weak features are observed in the 940–947 eV region, and no pronounced shake-up satellite structure characteristic of Cu^2+^-dominated species is present [26]. Because a monochromated Mg Kα source was used, contributions from Mg Kα_3,4_ source satellites are expected to be strongly suppressed [30]. These weak features were therefore not fitted as an independent Cu^2+^ component. Since the Cu 2p binding energies of Cu^+^ and Cu^0^ strongly overlap, the Cu 2p spectrum alone cannot distinguish these two states [31]. The Cu LMM Auger spectrum (Figure S3) shows a main feature at approximately 916.7 eV. Combined with the Cu 2p_3/2_ binding energy of 932.5 eV, the modified Auger parameter is approximately 1849.2 eV, supporting the assignment of Cu(I)/Cu_2_O [31]. This assignment is further supported by the Cu_2_O diffraction reflections observed by XRD. The persistence of a clear Cu signal further indicates that the Na_2_MoO_4_-containing surface layer is not a dense and fully continuous shell over the entire fiber surface. Instead, the deposited layer is likely porous or locally nonuniform, leaving portions of the underlying Cu_2_O-modified surface accessible to XPS detection. The Mo 3d spectrum (Figure 2f) is fitted with a Mo^6+^ doublet centered at approximately 232.14 and 235.23 eV, corresponding to Mo 3d_5/2_ and Mo 3d_3/2_, respectively. Possible Mo^5+^ and Mo^4+^ contributions were also examined; however, no clearly resolved additional doublet was supported by the measured spectral envelope and residual analysis. Therefore, no additional lower-valence Mo components were introduced in the final fitting [32].

### Electrochemical Performance

**Figure 3:**
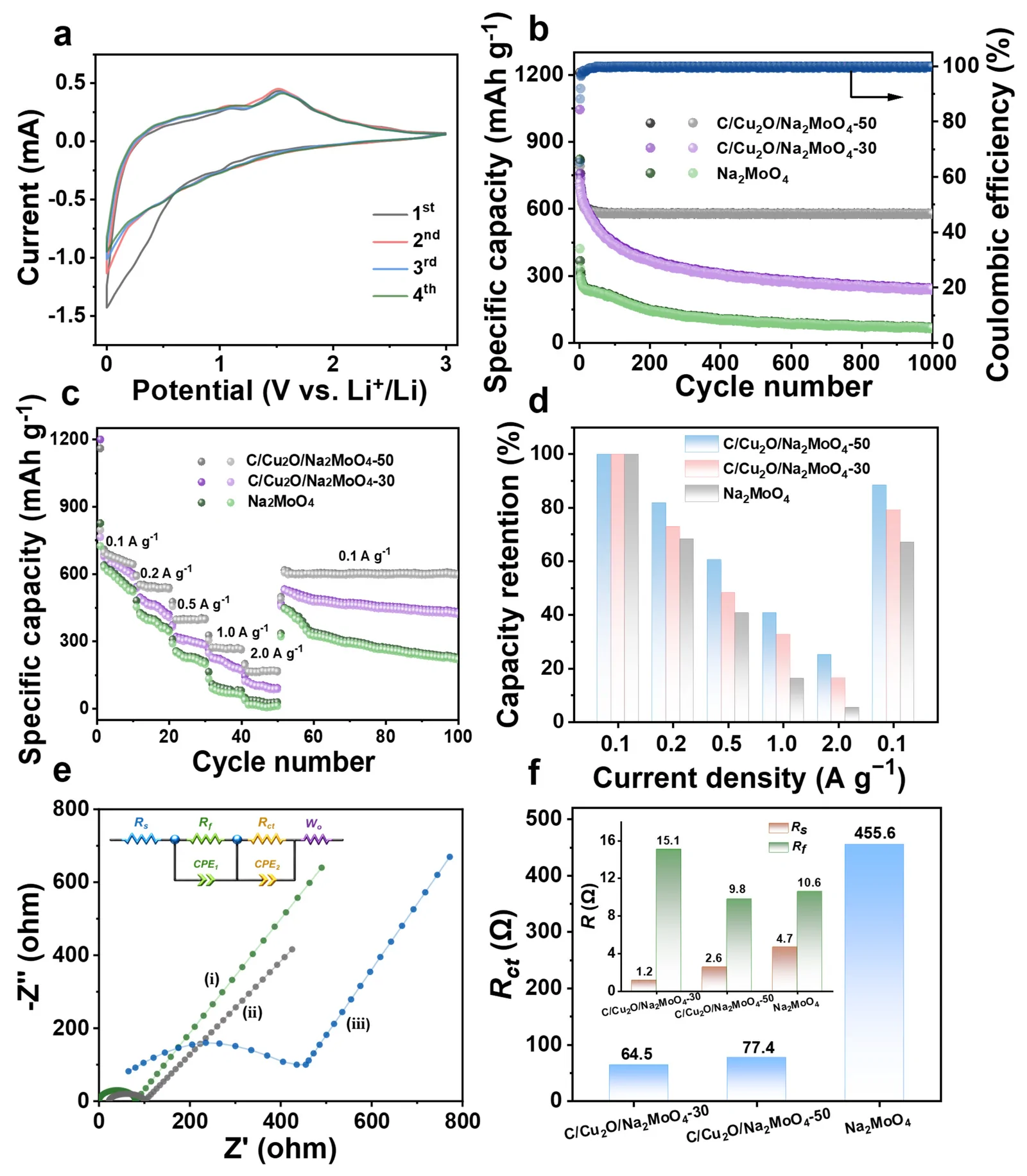
Electrochemical performance of the C/Cu_2_O/Na_2_MoO_4_ composite fibers and pristine Na_2_MoO_4_ electrode. (**a**) Cyclic voltammetry curves of C/Cu_2_O/Na_2_MoO_4_-50. (**b**) Long-term cycling performance of C/Cu_2_O/Na_2_MoO_4_-30, C/Cu_2_O/Na_2_MoO_4_-50, and Na_2_MoO_4_ electrodes at 0.5 A g^−1^, together with the Coulombic efficiency of C/Cu_2_O/Na_2_MoO_4_-50. (**c**) Rate capability of the three electrodes at different current densities. (**d**) Capacity retention under various current densities. (**e**) Nyquist plots of the (i) C/Cu_2_O/Na_2_MoO_4_-50, (ii) C/Cu_2_O/Na_2_MoO_4_-30, and (iii) Na_2_MoO_4_, electrodes with the equivalent circuit model shown in the inset. (**f**) Fitted impedance parameters of the electrodes; the blue bars represent *R_ct_*, while the inset shows *R_s_* and *R_f_*.

The electrochemical properties of the C/Cu_2_O/Na_2_MoO_4_ composite fibers were evaluated to understand the influence of hierarchical architecture and controllable Na_2_MoO_4_ loading on lithium-storage behavior. Figure 3a presents the first four CV curves of C/Cu_2_O/Na_2_MoO_4_-50 at 0.1 mV s^−1^. During the first cathodic curve, broad reduction features appear at approximately 1.0 and 0.7 V. The peak near 1.0 V is assigned to the reduction in Mo^6+^-containing Na_2_MoO_4_ species, while the lower potential broad peak is associated with further conversion reactions accompanied by SEI formation [6,10]. During the anodic process, the oxidation peaks near 1.1 and 1.6 V correspond to reoxidation of reduced Mo species, while the weak peak near 2.4 V is assigned to the reoxidation of metallic Cu toward Cu_2_O during the reverse conversion process. The corresponding conversion reaction can be described as [4,33]:

Cu_2_O + 2Li^+^ + 2e^−^ ↔ 2Cu + Li_2_O (1)

Compared with pristine Na_2_MoO_4_ in Figure S4, the second to fourth CV curves of C/Cu_2_O/Na_2_MoO_4_-50 show substantially greater overlap, demonstrating higher electrochemical reversibility after the initial activation cycle.

For the Na_2_MoO_4_ component, lithium storage proceeds through a multistep reduction and conversion process rather than simple Li^+^ insertion into an unchanged Na_2_MoO_4_ lattice. Previous studies have described the overall lithiation reaction of Na_2_MoO_4_ as follows [6,11]:

Na_2_MoO_4_ + 6Li^+^ + 6e^−^ → Mo + Na_2_O + 3Li_2_O (2)

The broad cathodic features in the CV profiles are therefore associated with the progressive reduction in Mo-containing species and the subsequent conversion reaction. After the initial activation process, the nearly overlapped CV profiles of the composite electrode indicate that the hierarchical carbon-supported architecture maintains stable electrochemical interfaces and facilitates reversible conversion reactions.

The long-term cycling behaviors of different electrodes were evaluated at a constant current density of 0.5 A g^−1^, as shown in Figure 3b. Pristine Na_2_MoO_4_ exhibits continuous capacity decay during prolonged cycling, with the reversible capacity gradually decreasing to only approximately 66.7 mAh g^−1^ after 1000 cycles. This poor cycling stability originates from the intrinsic low electrical conductivity of Na_2_MoO_4_ and the severe structural degradation associated with repeated conversion reactions, which leads to insufficient electrical contact and continuous loss of electrochemically active sites.

In contrast, both C/Cu_2_O/Na_2_MoO_4_-30 and C/Cu_2_O/Na_2_MoO_4_-50 composite fibers maintain much higher reversible capacities throughout cycling. The C/Cu_2_O/Na_2_MoO_4_-30 delivers initial discharge/charge capacities of 1043 and 716 mAh g^−1^, with an initial Coulombic efficiency of 68.7%. With the LbL cycle number increased to 50, C/Cu_2_O/Na_2_MoO_4_-50 reaches initial discharge/charge capacities of 1211 and 790 mAh g^−1^, with an initial Coulombic efficiency of 65.2%. Meanwhile, the relatively low initial Coulombic efficiencies are mainly associated with irreversible lithium consumption during the first lithiation process, including electrolyte decomposition, SEI formation, and partial irreversible conversion reactions. To reduce the first-cycle irreversible Li loss, pre-lithiation could be used to compensate for Li consumed by SEI formation and irreversible side reactions [34]. Electrolyte optimization, particularly through suitable film-forming additives such as fluoroethylene carbonate (FEC) or vinylene carbonate (VC), may further stabilize the SEI and suppress electrolyte decomposition during initial lithiation [35]. These strategies provide feasible routes for further improving the initial Coulombic efficiency of the composite electrode. The number of LbL cycles may also indirectly influence the initial interfacial reactions by changing the loading and surface coverage of the Na_2_MoO_4_-containing phase. The slightly lower ICE of C/Cu_2_O/Na_2_MoO_4_-50 (65.2%) than that of C/Cu_2_O/Na_2_MoO_4_-30 (68.7%) may reflect somewhat greater irreversible Li consumption associated with electrolyte decomposition, SEI formation, and the initial conversion of the higher-loaded Na_2_MoO_4_ phase. The higher reversible capacity of C/Cu_2_O/Na_2_MoO_4_-50 is maintained during prolonged cycling, indicating that the additional Na_2_MoO_4_ introduced by LbL assembly can still participate effectively in reversible lithium storage. After 1000 cycles, the reversible capacities of C/Cu_2_O/Na_2_MoO_4_-30, C/Cu_2_O/Na_2_MoO_4_-50 and pristine Na_2_MoO_4_ are approximately 240.8, 579.3 and 66.7 mAh g^−1^, respectively.

The electrochemical performance is not expected to increase monotonically with the number of LbL cycles. At relatively low cycle numbers, such as 10 or 20 cycles, the limited amount of deposited Na_2_MoO_4_ restricts the contribution of the conversion-active phase to the overall capacity. Increasing the LbL cycle number from 30 to 50 increases the Na_2_MoO_4_ loading and improves the reversible capacity of the 30-cycle and 50-cycle electrode. However, excessive LbL deposition can produce an overly thick and highly loaded Na_2_MoO_4_-containing layer, which may increase polarization and become mechanically less stable during repeated conversion reactions. Preliminary tests using substantially higher LbL cycle numbers showed poorer cycling reproducibility, indicating that further increasing the deposition cycles does not necessarily improve electrochemical performance. Therefore, the 30- and 50-cycle samples were selected as representative electrodes within the practically useful loading range, with the 50-cycle sample showing the best performance among the systematically evaluated samples. In this composition, the higher Na_2_MoO_4_ loading provides more electrochemically active sites, while the carbon scaffold restricts excessive structural degradation during repeated conversion reactions. The Cu_2_O-modified region provides an intermediate interface for assembling the Na_2_MoO_4_-containing phase and helps maintain contact between the active components and carbon framework.

The rate capability of the electrodes was further evaluated by comparing the capacity retention under different current densities (Figure 3c,d). As the current density increased from 0.1 to 2.0 A g^−1^, all electrodes exhibited a gradual decrease in capacity retention due to enhanced polarization and insufficient reaction kinetics at high-rate conditions. At the initial current density of 0.1 A g^−1^, all electrodes were normalized to 100% capacity retention. When the current density increased to 0.2 A g^−1^, the capacity retention values of C/Cu_2_O/Na_2_MoO_4_-30, C/Cu_2_O/Na_2_MoO_4_-50, and Na_2_MoO_4_ decreased to 73.00%, 81.81%, and 68.34%, respectively. With further increasing the current density to 2.0 A g^−1^, C/Cu_2_O/Na_2_MoO_4_-50 still maintained a capacity retention of 25.20%, which was higher than those of C/Cu_2_O/Na_2_MoO_4_-30 (16.36%) and pristine Na_2_MoO_4_ (5.36%). These results indicate that the composite fibers possess improved rate capability under high current densities.

After the current density was returned to 0.1 A g^−1^, the C/Cu_2_O/Na_2_MoO_4_-50 electrode recovered 88.52% of its initial capacity, whereas C/Cu_2_O/Na_2_MoO_4_-30 and Na_2_MoO_4_ recovered only 79.18% and 67.14%, respectively. The higher recovery of the composite electrodes indicates a smaller irreversible loss of accessible capacity after high-rate cycling. Notably, C/Cu_2_O/Na_2_MoO_4_-50 maintains the highest capacity retention throughout the rate test despite its larger Na_2_MoO_4_ loading.

Electrochemical impedance spectroscopy was used to compare the interfacial reaction kinetics of the three electrodes. The Nyquist plots are shown in Figure 3e, and the corresponding fitted resistance parameters are summarized in Figure 3f. Pristine Na_2_MoO_4_ exhibits the largest charge transfer resistance (*R_ct_*) of 455.6 Ω, whereas the *R_ct_* values of C/Cu_2_O/Na_2_MoO_4_-30 and C/Cu_2_O/Na_2_MoO_4_-50 decrease to 64.5 and 77.4 Ω, respectively. Increasing the LbL cycles from 30 to 50 raises *R_ct_* by only 12.9 Ω, showing that the additional Na_2_MoO_4_ surface layer introduces a measurable but limited increase in interfacial resistance. Even after this increase, the *R_ct_* of C/Cu_2_O/Na_2_MoO_4_-50 remains approximately 83% lower than that of pristine Na_2_MoO_4_. More importantly, C/Cu_2_O/Na_2_MoO_4_-50 delivers substantially higher cycling and rate retention than the 30-cycle electrode despite its slightly larger *R_ct_*. The electrochemical advantage of the 50-cycle sample therefore comes from adding more electrochemically usable Na_2_MoO_4_ while keeping the charge transfer resistance far below that of the unsupported Na_2_MoO_4_ electrode.

As summarized in Table 1, C/Cu_2_O/Na_2_MoO_4_-50 exhibits markedly improved long-term cycling performance compared with previously reported Na_2_MoO_4_-based anodes. Among representative conversion-type electrodes, including Fe_3_O_4_/C, Co_3_O_4_/C, and MoS_2_/C composites, the present electrode retains a similar or higher reversible capacity over a substantially extended cycling period. Compared with carbon-based and Si/C anodes, C/Cu_2_O/Na_2_MoO_4_-50 also maintains a high reversible capacity after 1000 cycles.

These comparisons show that the introduction of Na_2_MoO_4_ onto the hierarchical cotton-derived carbon framework increases the long-term capacity beyond that of previously reported Na_2_MoO_4_-based and cotton-derived carbon electrodes, while maintaining cycling durability comparable to several representative conversion-type and Si/C anodes.

**Figure 4:**
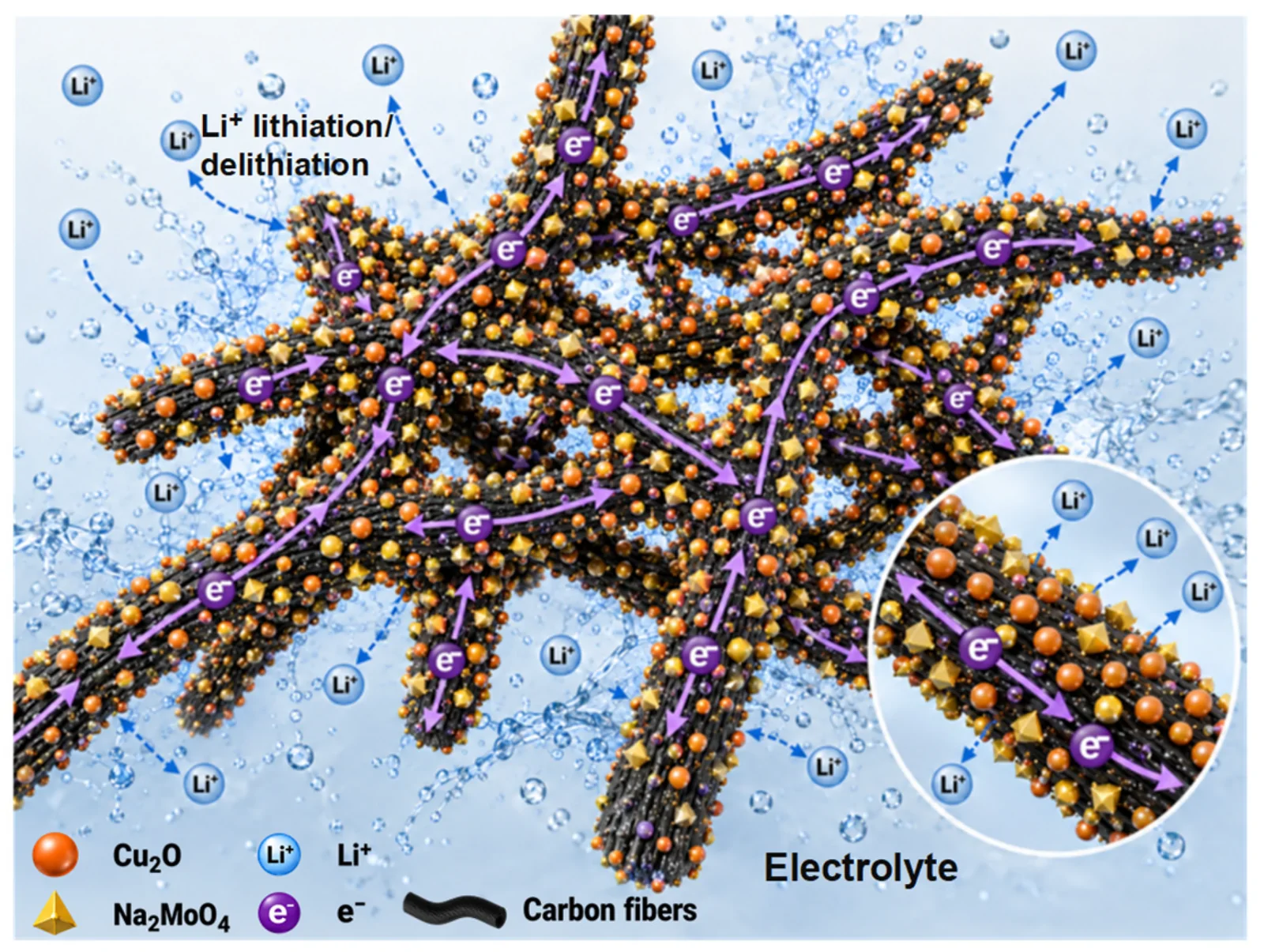
Schematic illustration of the lithium-storage mechanism of the hierarchical C/Cu_2_O/Na_2_MoO_4_ composite electrode.

The proposed lithium-storage mechanism of the hierarchical C/Cu_2_O/Na_2_MoO_4_ composite fibers is illustrated in Figure 4. During cycling, Li^+^ ions migrate through the electrolyte to the surface-confined active phases, whereas electrons are transported through the interconnected cotton-derived carbon network [41,42,43]. Na_2_MoO_4_ undergoes multistep reduction and conversion of Mo species, while Cu_2_O participates in the reversible Cu_2_O/Cu conversion reaction. The surface location of the active phase gives Li^+^ a short radial transport path from the electrolyte to the reaction region, while the continuous carbon fibers provide a separate long-range pathway for electron conduction. The active phase therefore does not rely on electron transport through bulk Na_2_MoO_4_ particles. During conversion reactions, the volume changes occur in the inorganic active components. The fibrous carbon scaffold surrounds these components with a continuous conductive framework, so the conversion products remain in electrical contact with the electrode network during repeated lithiation and delithiation. Cu_2_O performs two functions. It participates in lithium storage through the Cu_2_O/Cu conversion reaction and provides the inorganic surface used for subsequent LbL deposition. Sequential PDDA and MoO_4_^2−^ adsorption distributes the Na_2_MoO_4_-containing phase along the fiber surface. Increasing the number of LbL cycles therefore increases the thickness and amount of this surface active layer rather than converting the electrode into a bulk Na_2_MoO_4_ particle assembly. 

Overall, the improved lithium-storage behavior of the composite fibers results from the coordinated design of active-phase distribution and conductive architecture. The LbL-assembled Na_2_MoO_4_ layer increases the utilization of conversion-active species, while the cotton-derived carbon framework maintains efficient electron transport and structural integrity during repeated conversion reactions. This architecture demonstrates that controlled interfacial assembly is an effective strategy for improving the reversibility of conversion-type electrodes.

From a practical perspective, the use of cotton-derived fibers and Cu_2_O provides a relatively simple material basis for scale-up. For the present 30–50-cycle LbL process, future optimization should focus on increasing the amount deposited per cycle, thereby reducing the number of repeated adsorption and rinsing steps required to reach the target Na_2_MoO_4_ loading. Continuous fiber processing and solution recirculation may further improve the efficiency of large-scale preparation.

## 4. Conclusions

In summary, hierarchical C/Cu_2_O/Na_2_MoO_4_ composite fibers were successfully constructed by integrating a cotton-derived micro- and nanofibrous carbon framework with Cu_2_O interfacial modification and controllable Na_2_MoO_4_ assembly. The resulting material combines an interconnected carbon fiber network and contains a Cu_2_O-modified surface covered by a controllable Na_2_MoO_4_-containing layer. When used as anodic materials for lithium-ion batteries, the C/Cu_2_O/Na_2_MoO_4_-50 composite fibers exhibit improved reversible capacity, enhanced rate capability, and superior cycling stability compared with pristine Na_2_MoO_4_. The improvement results from placing a larger amount of conversion-active Na_2_MoO_4_ directly on an electronically continuous carbon framework, which shortens the Li^+^ transport distance to the active surface and maintains electron conduction during repeated conversion reactions. Cu_2_O additionally provides both conversion activity and the interfacial surface required for LbL deposition. The present work provides insights into the design of biomass-derived hierarchical electrodes and highlights the potential of interface-controlled assembly strategies for constructing advanced conversion-type energy-storage materials.

## Figures and Tables

**Table 1:** Comparison of the electrochemical performance of C/Cu_2_O/Na_2_MoO_4_-50 with representative Na_2_MoO_4_-based, conversion-type, Si/C, and carbon-based anodes.

Material	Structural Strategy	Cycling Performance	Current Density	Cycles	Reference
Carbon-coated *α*-Na_2_MoO_4_	Na_2_MoO_4_ nanoplates/carbon coating	350 mAh g^−1^	30 mA g^−1^	30	[11]
Na_2_MoO_4_/TiO_2_	Cellulose-templated nanotubes + LbL	180.22 mAh g^−1^	0.1 A g^−1^	200	[6]
Fe_3_O_4_/C	conversion oxide/carbon microspheres	500 mAh g^−1^	1 C	300	[36]
C-doped Co_3_O_4_	porous conversion oxide/carbon	539.2 mAh g^−1^	1 A g^−1^	500	[37]
1T-MoS_2_/C_0.6_	layered sulfide/carbon composite	570 mAh g^−1^	0.1 A g^−1^	500	[38]
Si@C	alloying-type Si/carbon	583.3 mAh g^−1^	1 A g^−1^	500	[39]
Cotton-derived hollow carbon	biomass-derived carbon	450 mAh g^−1^	0.1 A g^−1^	300	[40]
C/Cu_2_O/Na_2_MoO_4_-50	Cotton derived C + Cu_2_O + LbL Na_2_MoO_4_	579.3 mAh g^−1^	0.5 A g^−1^	1000	this work

## Data Availability

The original contributions presented in this study are included in the article and Supplementary Material. Further inquiries can be directed to the corresponding author.

## Supplementary Materials

The following supporting information can be downloaded at: https://www.mdpi.com/article/10.3390/nano16181160/s1, Figure S1: XPS survey spectrum of the C/Cu_2_O/Na_2_MoO_4_-50 composite fibers, confirming the coexistence of C, O, Cu, Mo, and Na elements; Figure S2. High-resolution Na 1s XPS spectrum and fitted components of C/Cu_2_O/Na_2_MoO_4_-50; Figure S3. Cu LMM Auger spectrum of C/Cu_2_O/Na_2_MoO_4_-50, showing the characteristic Cu(I)/Cu_2_O Auger feature centered at approximately 916.7 eV; Figure S4. CV curves of the pristine Na_2_MoO_4_ electrode at a scan rate of 0.1 mV s^−1^ within a potential range of 0.01–3.0 V (vs. Li^+^/Li); Table S1. Binding energies, FWHM values, and fitting constraints for the high-resolution XPS spectra.
