# Supplementary material for: Layer-by-Layer Assembly of Hierarchical Cotton-Derived C/Cu2O/Na2MoO4 Composite Fibers as Anodes for Lithium-Ion Batteries
Source: Nanomaterials (Basel). 2026 Sep 15;16(18):1160. doi: 10.3390/nano16181160 (PMC13610098; doi:10.3390/nano16181160)
Supplement: Supplementary file 1 [file nanomaterials-16-01160-s001.zip › nanomaterials-4558724-supplementary.pdf]

Supplementary Materials

Layer-by-Layer Assembly of Hierarchical Cotton-Derived $C/Cu_2O/Na_2MoO_4$ Composite Fibers as Anodes for Lithium-Ion Batteries

Shun Li[*a,b], Jidong Zhang[b], Shuqi Li[a], Ying Guan[a], Guijin He[b*]

[a] *School of Chemical and Biological Engineering, Hangzhou Polytechnic University, Hangzhou 311300, China*

[b] *Department of Chemistry, Zhejiang University Hangzhou, Zhejiang 310027, China*

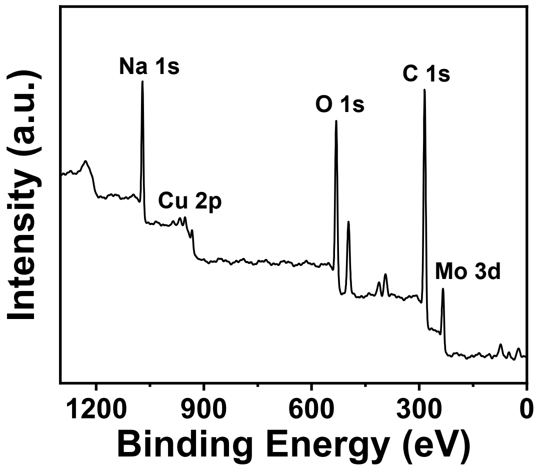

Figure S1. XPS survey spectrum of the $C/Cu_2O/Na_2MoO_4$-50 composite fibers, confirming the coexistence of C, O, Cu, Mo, and Na elements.

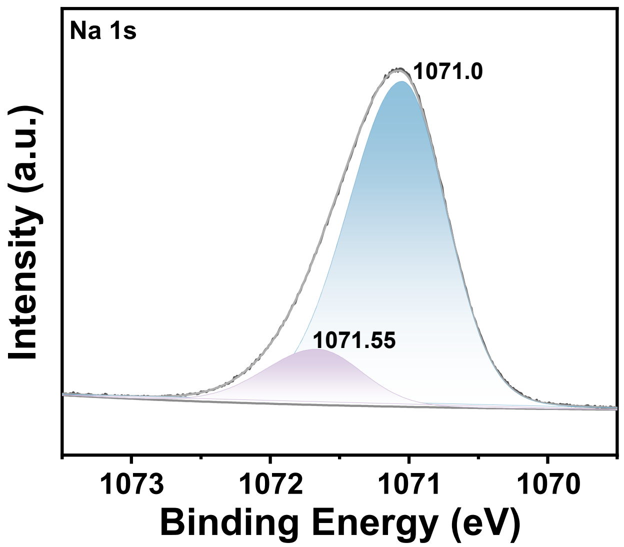

Figure S2. High-resolution Na 1s XPS spectrum and fitted components of $C/Cu_2O/Na_2MoO_4$-50.

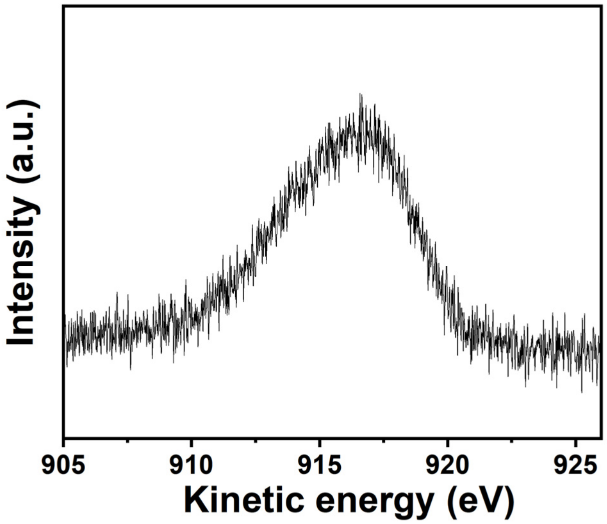

Figure S3. Cu LMM Auger spectrum of $C/Cu_2O/Na_2MoO_4$-50, showing the characteristic Cu(I)/Cu_2O Auger feature centered at approximately 916.7 eV.

Table S1. Binding energies, FWHM values, and fitting constraints for the high-resolution XPS spectra.

XPS region	Assignment	Binding energy (eV)	FWHM (eV)	Fitting constraints / notes
C 1s	C–C/C=C	284.80	1.81	Charge-reference peak fixed at 284.8 eV; Shirley background; common pseudo-Voigt line-shape
C 1s	C–O	286.50	1.74	Center constrained to the assigned C–O region; Shirley background; common pseudo-Voigt line-shape
C 1s	C=O / O–C=O	288.80	2.39	Center constrained to the carbonyl/carboxyl region; Shirley background; no extra C 1s component introduced
O 1s	Metal–O (combined Cu–O/Mo–O contribution)	530.50	2.70	Center constrained to lattice-metal–oxygen region; Shirley background; assignment kept non-exclusive
O 1s	Surface O / C–O	532.40	1.49	Center constrained to surface-oxygen/C–O region; Shirley background
O 1s	OH / adsorbe d H_2O	533.80	2.20	Center constrained to high-binding-energy surface species; Shirley background
Cu 2p	Cu $2p_{3/2}$	932.50	2.86	Main Cu 2p peak only; Shirley background; no Cu^+/Cu^0 deconvolution from Cu 2p alone
Cu 2p	Cu $2p_{1/2}$	952.30	2.93	Spin–orbit separation ≈ 19.8 eV; comparable FWHM; weak 940–947 eV features not fitted as an independent Cu^{2+} state
Mo 3d	Mo^{6+} $3d_{5/2}$	232.14	1.35	Shirley background; same line-shape for the doublet; area ratio con strained to ~3:2
Mo 3d	Mo^{6+} $3d_{3/2}$	235.23	1.36	Spin–orbit separation = 3.09 eV; area ratio $3d_{5/2}$:$3d_{3/2}$ ≈ 3:2; Mo^{5+}/Mo^{4+} not retained

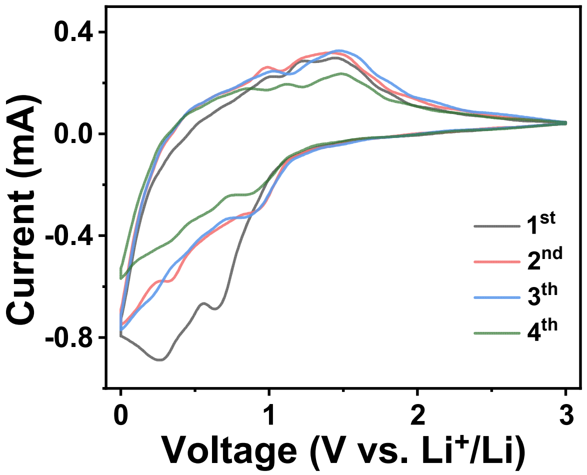

Figure S4. CV curves of the pristine Na_2MoO_4 electrode at a scan rate of 0.1 mV s^{-1} within a voltage window of 0.01–3.0 V (vs. Li^+/Li).
